# Flow Cytometric Quantification of Mitochondrial Properties: A High-Throughput Approach for Single Organelle Analysis

**DOI:** 10.3390/ijms26125481

**Published:** 2025-06-07

**Authors:** Andrew J. Piasecki, Hannah C. Sheehan, Jonathan L. Tilly, Dori C. Woods

**Affiliations:** Department of Biology, Northeastern University, Boston, MA 02115, USA

**Keywords:** flow cytometry, mitochondria, organelle sorting, fluorescence-activated mitochondria sorting, extracellular vesicle sorting

## Abstract

Recent advances in flow cytometry facilitate the detection of subcellular components, such as organelles and vesicles. Fluorescence-activated mitochondria sorting (FAMS) is a flow cytometry-based technique that allows for quantitative analysis and sorting of mitochondria as individual organelles from various tissues and in vitro cell culture. This manuscript details three novel applications of this technique to study mitochondrial function on an organelle-specific level, which is not possible with other approaches. Specifically, we detail the further development and versatility of this nanoscaled flow cytometry approach, including assays to quantitatively assess mitochondrial subpopulations, mitochondrial protein translocation, and both free-floating and EV-encapsulated secreted mitochondria. We demonstrate a multi-parameter quantitative assay for the analysis of mitochondrial autophagy using antibodies targeting the proteins PINK1 and Parkin corresponding to ΔΨ_M_ and further show how these can be assessed for mtDNA content on a single organelle level. Further, we establish parameters for the size and surface marker-based analysis of EVs, many of which contain identifiable and respiring mitochondria, as well as free-floating respiratory-competent mitochondria. These results display the versatility of nanoscaled flow cytometry in terms of both sample input and target organelle and provide an important methodological means for the quantitative assessment of mitochondrial features.

## 1. Introduction

While flow cytometry has been widely used for cell analysis, recent advancements in technology have made it possible to analyze much smaller particles, including organelles, opening new possibilities for the study of the nuances of intracellular dynamics. The field of mitochondrial research stands to particularly benefit from these advancements, given both their importance in multiple cellular functions and their great range of diversity. With the roles mitochondria play within the cell rapidly expanding beyond their more well-known function as energy producers, finding new and innovative ways of assessing these functions is paramount. Previously, we developed a multi-parametric nanoscaled flow cytometry platform termed FAMS (fluorescence-activated mitochondria sorting) to analyze and isolate mitochondria as individual entities [1]. Single organelle analysis is particularly useful in mitochondrial research because mitochondria are inherently heterogeneous, with differences in morphology, cristae density, membrane potential (ΔΨ_M_), proteomic landscape, and mitochondrial DNA (mtDNA) content existing not only between different cell types but even within a single cell [2,3,4,5,6]. This heterogeneity has led to the classification of mitochondria into subpopulations to further refine their role as signaling hubs within cells, carrying out diverse functions, including processes such as energy production, hormone biosynthesis, heme biosynthesis, thermodynamics, and regulation of apoptosis [7,8,9]. We have previously shown that single mitochondria can be analyzed and sorted via FAMS based on characteristics such as size, ΔΨ_M_, and protein expression, which enables the isolation of distinct mitochondrial subpopulations that may differ in their physiology and responses to stimuli or stressors. For example, mitochondria with different ΔΨ_M_ values may reflect varying stages of dysfunction or activation, which could be related to energy production, apoptotic priming, or stress responses [1].

The ability to analyze and sort single mitochondria via flow cytometry has specific advantages over traditional techniques, such as mitochondrial staining of live cells via fluorescence microscopy or bulk assays (e.g., PCR or immunoblotting), which typically lack the ability to analyze individual mitochondria or quantitatively discern subpopulations within a larger population. By comparison, the organelle-specific data obtained through flow cytometry allows for the simultaneous quantitative evaluation of multiple parameters on an individual level, significantly refining the ability to detect subtle changes in mitochondrial activity, such as variations in ΔΨ_M_, reactive oxygen species (ROS) generation, and metabolic shifts, which may be obscured in bulk assays. Additionally, flow cytometry can be adapted to study specific mitochondrial markers or probe for the presence of mitochondria in different locations [e.g., mitochondria in blood or extracellular vesicles (EVs)], enabling insights into cellular communication, mitochondrial trafficking, and potential contributions to pathologies like cancer metastasis or neurodegenerative diseases. Herein, we expand the utility of flow cytometry-based mitochondrial analysis for the purpose of directly evaluating samples on a per-organelle basis, allowing for the partition of the mitochondrial pool into subpopulations. This includes the simultaneous multi-target assessment of protein expression and ΔΨ_M_ of individual mitochondria entering the mitophagy pathway. Previously, this process could only be resolved at the cellular level, where aggregate values from the total mitochondrial population were ascertained. Additionally, FAMS was used to confirm the presence of cell-free, respiratory-competent mitochondria in mouse plasma, which had been previously theorized to exist, but technical limitations prevented a clear demonstration of their ability to respire. Finally, both free-floating and EV-encapsulated secreted mitochondria were detected in THP-1 macrophage media, displaying both the ability of FAMS to detect and isolate organelles other than mitochondria and investigate the relationship between them.

## 2. Results

### 2.1. Development of a Biological Assay for the Quantitative Assessment of Mitophagy in Individual Mitochondria

The autophagic removal of mitochondria, termed “mitophagy”, is a vital mechanism for maintaining mitochondrial quality and quantity as well as the regulation of cellular function. In damaged or dysfunctional mitochondria, the serine/threonine–protein kinase, phosphatase and tensin homolog (PTEN)-induced putative kinase 1 (PINK1), is stabilized on the outer mitochondrial membrane, where it then recruits and activates the E3 ubiquitin ligase, Parkin. Initially, PINK1 accumulation was thought to occur specifically in response to a drop in ΔΨ_M_; however, more recently, studies have suggested ΔΨ_M_ may not be the only trigger [10]. One such example would be the misfolding of mitochondrial proteins shown to trigger PINK1 activation without a drop in ΔΨ_M_ [11,12]. Therefore, it is important to have an assay to assess PINK1 and Parkin without utilizing membrane uncouplers or alternative agents that impact ΔΨ_M_ as the sole means of validation [13].

To increase the rate of mitophagy without impacting baseline ΔΨ_M_ through the use of electron transport chain uncouplers, we generated an immortalized fibroblast cell line from *PolG* mutant mice, which harbor a mutation in the mitochondrial DNA (mtDNA) polymerase γ (*PolG*), which is responsible for the proofreading and base/mismatch repair of the mitochondrial genome [14]. This greatly reduces the mtDNA proofreading capability, thereby increasing the rate of mitophagy in these cells through an elevated rate of mtDNA mutation [15,16,17]. The inherent increase in mitophagy in *PolG* mutant cells allows for the labeling and study of mitophagy-related proteins without further mitochondrial manipulation or cellular insult.

**Figure 1:**
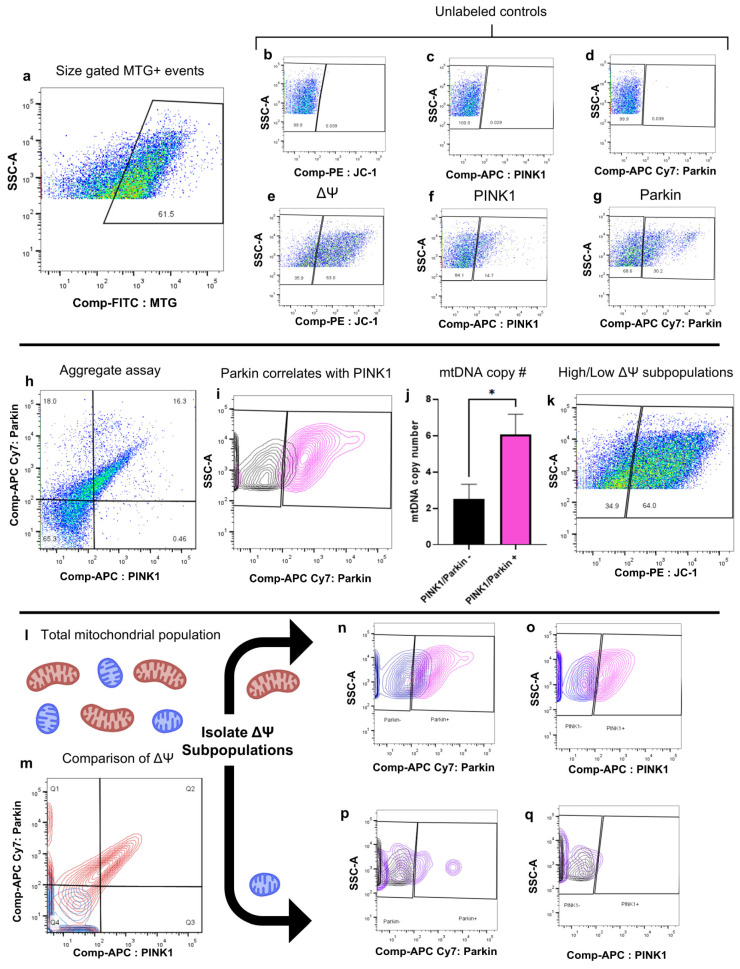
Assay for the quantitative assessment of mitophagy in individual mitochondria without the use of membrane uncouplers. (**a**) Example gate for the detection of mitochondria. MTG-stained samples, which had been gated for size, as described in Figure 1, are analyzed for FITC fluorescence to discern labeled mitochondria from non-fluorescent background events of similar sizes. (**b**–**d**) Negative control samples: These samples were stained only with MTG, and these MTG+ events were used to establish subsequent gates for ΔΨ_M_(PE), PINK1 expression (APC), and Parkin (APC-Cy7). (**e**–**g**) Stained samples used to confirm gates drawn in b-d and used to apply compensation between channels to eliminate spillover between similar fluorophores, particularly APC and APC-Cy7. As above, these sample gates are applied to events that have been previously gated by size and MTG fluorescence to confirm they are mitochondria. (**h**) Example of global PINK1 and Parkin expression on mitochondria isolated from PolG fibroblasts. A total of 65.3% of mitochondria (SEM = 1.30%, *n* = 3) were found to express neither PINK1 nor Parkin, 16.1% of mitochondria (SEM = 1.43%, *n* = 3) expressed both PINK1 and Parkin, 18.23% of mitochondria (SEM = 0.67%, *n* = 3) expressed only Parkin, and 0.46% of mitochondria (SEM = 0.04%, *n* = 3) expressed only PINK1. (**i**) Parkin expression on mitochondria that expressed (pink) or did not express (black) PINK1. Of PINK1-positive mitochondria, 95.23% (SEM = 0.69%, *n* = 3) were also positive for Parkin, and of PINK1-negative mitochondria, 80.7% (SEM = 0.71%, *n* = 3) were also negative for Parkin. (**j**) Comparison of mtDNA copy number (#) on the basis of PINK1/Parkin expression. Mitochondria negative for PINK1/Parkin (black, mean = 1.12, SEM = 0.40, *n* = 3) had significantly lower mtDNA copy number than PINK1/Parkin-positive mitochondria (pink, mean = 6.07, SEM = 1.11, *n* = 3, * indicates statistical significance *p* = 0.013). (**k**) JC-1 staining of ΔΨ_M_ across the total mitochondrial population. (**l**) Schematic depicting the isolation of high (red) and low (blue) ΔΨ_M_ mitochondria. (**m**) Comparison of PINK1/Parkin expression in high (red) and low (blue) high ΔΨ_M_ mitochondrial subpopulations. (**n**) Comparison of Parkin expression on PINK1+ (pink) and PINK1- (blue) in high ΔΨ_M_ mitochondria. (**o**) Comparison of PINK1 expression on Parkin+ (pink) and Parkin- (blue) in high ΔΨ_M_ mitochondrial subpopulations. (**p**) Comparison of Parkin expression on PINK1+ (pink) and PINK1- (blue) in low ΔΨ_M_ mitochondria. (**q**) Comparison of PINK1 expression on Parkin+ (pink) and Parkin- (blue) in low ΔΨ_M_ mitochondrial subpopulations.

To evaluate mitochondrial-associated PINK1 and Parkin, cell-lysate samples isolated from *PolG* mutant cells were first gated for events that fell within a 0.2–2 μm mitochondrial size range [2]. Event size was detected using FSC-PMT values using sizing beads (0.22 μm, 0.45 μm, 1 μm, 2 μm, and 4 μm) as reference points, as described in methods Section 4.3. Once events were determined to be within the mitochondrial size range, MitoTracker™ Green FM (MTG, a mitochondria-specific dye that fluoresces in the FITC channel) was then used to differentiate mitochondria from any cellular debris that happened to fall within the size gate (Figure 1a). With the mitochondrial signal from the samples isolated, additional markers were used to determine ΔΨ_M_ as well as PINK1 and Parkin expression (Figure 1b–g). ΔΨ_M_ was evaluated using JC-1, a cationic dye that aggregates inside mitochondria with a high ΔΨ_M_. The aggregation of JC-1 causes the fluorescence profile to shift from emitting in the FITC channel (non-aggregated) to emitting in the PE channel (aggregated). Importantly, mitochondria with low ΔΨ_M_ do not always contain non-aggregated JC-1; therefore, the inclusion of MTG ensures that all mitochondria are labeled. The expression of PINK1 and Parkin was detected using antibodies directly conjugated to a fluorophore: APC:PINK1 and APC-Cy7/Parkin. Single-color controls were used to generate a compensation matrix to eliminate any potential spillover between the APC and APC-Cy7 channels. Once compensation was applied, events that fell within both the size gate and MTG gate (requirements to be identified as a mitochondrion) could then be evaluated for the expression of PINK1 and Parkin.

**Figure 2:**
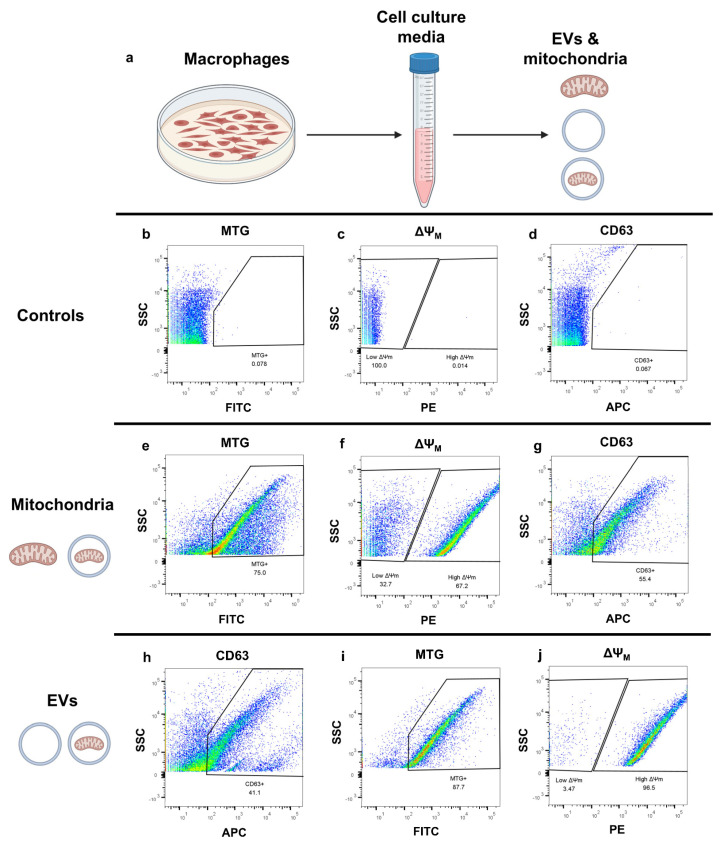
Flow cytometric analysis of excreted mitochondria and EVs in THP-1 macrophage media. (**a**) Schematic summarizing experimental workflow. (**b**–**d**) Negative controls for MTG (FITC), JC-1 (PE), and CD63 antibody (APC). Samples had been previously gated for size. MTG gate is the parent gate for ΔΨ_M_ and CD63 plots. (**e**) Identification of mitochondria. Events that fell within the size gate were assessed for FITC fluorescence. Events that met both the size criteria and were MTG+ were identified as mitochondria. (**f**–**g**) Evaluation of ΔΨ_M_ and CD63 expression in mitochondria (as determined by size gate and MTG stain) isolated from THP-1 media. A total of 66.4% of mitochondria (*n* = 3, SEM = 3.4) had a high ΔΨ_M_, and 50.7% of mitochondria (*n* = 3, SEM = 3.31) were positive for CD63. As CD63 is an EV marker and is not found on mitochondria, mitochondrial events that were CD63+ were interpreted to be mitochondria enclosed inside EVs. (**h**) Resetting the gating tree to first identify EVs (via CD63 expression). To identify EVs in the sample independent of mitochondrial detection, unlike previous panels, CD63 expression was assessed prior to any other gating (including size). This CD63+-positive gate serves as the parent gate for the remaining populations in the figure, as it positively identifies these events as EVs. (**i**) Mitochondrial stain of EVs. CD63+ events were gated based on mitochondrial size and then assessed on the FITC fluorescence channel to identify mitochondria via MTG staining. A total of 91.7% of the detected EVs (*n* = 3, SEM = 3.20) were found to contain mitochondria. (**j**) Assessment of ΔΨ_M_ in mitochondria enclosed within EVs. A total of 96.3% of mitochondria (*n* = 3, SEM = 1.19) found within EVs were found to have a high ΔΨ_M_.

Evaluating the mitochondrial population as a whole, 65.3% ± 1.30% (*n* = 3) of mitochondria were negative for PINK1 and Parkin localization, while 16.1% ± 1.43% (*n* = 3) expressed both PINK1 and Parkin (Figure 1h), indicative of activated mitophagy. Of the remaining mitochondria, 18.23% ± 0.67% (*n* = 3) expressed only Parkin, and only 0.46% ± 0.04% (*n* = 3) were labeled with only PINK1. These data indicate that, as anticipated, when PINK1 is present, Parkin is generally present as well; the inverse of this is not true, as Parkin expression was not a strong predictor of PINK1 expression (Figure 2i). PINK1/Parkin double-positive mitochondria were found to have a significantly increased average mtDNA count per organelle (mean = 6.07, SEM = 1.11, *n* = 3, *p* = 0.013) compared to PINK1/Parkin double-negative mitochondria (mean = 1.12, SEM = 0.40, *n* = 3) (Figure 1j). Mitochondrial subpopulation analysis was then conducted by independently evaluating high and low ΔΨ_M_ mitochondria (Figure 1k–m). High ΔΨ_M_ mitochondria comprised 63.9% ± 1.00% (*n* = 3) of the total mitochondrial population and showed similar patterns of PINK1 and Parkin interaction, with Parkin present on 96.2% ± 0.66% (*n* = 3) of high ΔΨ_M_ mitochondria labeled with PINK1 (Figure 1n), a notably stronger correlation than the presence of PINK1 on mitochondria labeled with Parkin (53.4% ± 2.88, *n* = 3, Figure 1o). Conversely, low ΔΨ_M_ mitochondria comprised 35.03% ± 1.07% (*n* = 3) of the total mitochondrial population and did not have similar rates of PINK1/Parkin colocalization compared to the overall mitochondrial population. Low to no PINK1/Parkin colocalization was observed in this subpopulation, supporting the recent evidence that initiation of endogenous mitophagy may not require a drop in ΔΨ_M_ as previously thought (Figure 1p,q).

### 2.2. Flow Cytometry-Based Assessment of Mitochondria-Containing Extracellular Vesicles in THP1 Macrophages

Recently, macrophages were shown to communicate with both healthy and cancerous tissue and are known to exude EVs [18,19]. Furthermore, vesicles produced by mesenchymal stem cells and other cell types have been shown to contain mtDNA [20,21], raising the question as to whether macrophages also secrete these vesicles and whether they contain intact mitochondria in addition to mtDNA. To make this determination, human THP-1 macrophages differentiated in vitro from THP-1 monocytes were cultured for 24 h prior to the collection of the spent media to test for the presence of mitochondria-containing EVs using FAMS (Figure 2a). The spent media was stained with MTG to detect mitochondria as well as JC-1 to assess ΔΨ_M_. EV detection was accomplished with an anti-CD63 antibody, as CD63 is present on the surface of EVs and is commonly used as a labeling marker. Both mitochondria and EVs were determined to be present after comparison to unstained controls (Figure 2b–d). Of all detected mitochondria, regardless of whether they were enclosed in an EV, 66.4% (*n* = 3, SEM = 3.4) displayed a high ΔΨ_M_, which suggests that these organelles would be respiratory-competent (Figure 2e,f). Of all the MTG+ events detected in the sample, 50.7% (*n* = 3, SEM = 3.31) were also positive for CD63 (Figure 2g), indicating that these mitochondria were packaged inside of vesicles, as described previously [22,23].

Conversely, our data reveal that 91.7% (*n* = 3, SEM = 3.20) of CD63+ events that fell within the mitochondrial size range were also positive for mitochondrial markers (Figure 2h,i) and that 96.3% (*n* = 3, SEM = 1.19) of those mitochondria maintained a high ΔΨ_M_ (Figure 2j). It is important to note that these samples were prepared using centrifugation speeds optimized for mitochondrial isolation, so the results shown here are not necessarily reflective of the total EV pool initially present in the media, as any vesicles that were substantially heavier or lighter than the average mitochondrion were likely lost during sample prep. However, our results do confirm the presence of mitochondria packaged within EVs that were excreted by cells.

### 2.3. Flow Cytometry-Based Analysis of Intact, Cell-Free Mitochondria from Blood

**Figure 3:**
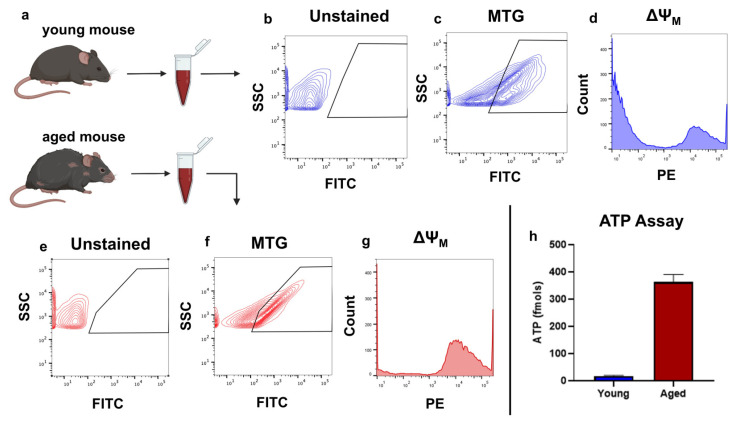
Detection of intact, cell-free mitochondria in the blood plasma of young (13 weeks) and aged (18 months) mice. (**a**) Schematic of blood collection. (**b**,**c**) Detection of mitochondria in young mouse blood plasma. After size gating, samples were assessed for MTG. Compared to the unstained control, mitochondria were identified in young mouse blood plasma. (**d**) ΔΨ_M_ of cell-free mitochondria in young mice. Some cell-free mitochondria were shown to have a high ΔΨ_M_, suggesting the ability to actively respire. (**e**–**g**) Identical analysis to b-d but with aged mouse plasma instead of young mouse plasma. (**h**) ATP assay of FAMS collected mitochondria from blood plasma. Aged mice averaged 364.4 fmol of ATP production (*n* = 3), and young mice averaged 18.0 fmol of ATP production. While the reason for this difference in ATP production is open to interpretation, it is sufficient to confirm the presence of respiratory-competent mitochondria in both young and aged mouse plasma.

Recent studies have identified intact, cell-free mitochondria in the plasma of cancer patients [24]. In healthy individuals, previous studies have identified circulating mtDNA in plasma but did not confirm the presence of intact organelles [25,26]. Interestingly, the abundance and impact of the detected mtDNA copies from plasma vary with age [27]. To determine whether plasma from healthy individuals contains intact, cell-free mitochondria and whether these mitochondria were able to maintain their ΔΨ_M_, blood was collected from both young (13 weeks old) and aged (18 months old) female C57BL/6 mice (Figure 3a). Samples were then centrifuged to isolate blood plasma and stained with MTG to allow for the detection of mitochondria. FAMS analysis corroborated the reports that there were, in fact, cell-free, respiratory-competent mitochondria present in the blood plasma, not just mtDNA molecules (Figure 3b–h). Both young and aged samples stained positively for MTG compared to unstained controls (Figure 3b,c,e,f). However, this finding begs the question of whether these mitochondria are intact or damaged. FAMS analysis concluded that in both young and aged mice, some mitochondria maintained a high ΔΨ_M_, which requires both the inner and outer mitochondrial membranes to be intact (Figure 3d,g). To further test functionality, cell-free mitochondria were collected from the plasma via FAMS and assayed for ATP synthesis, which confirmed that at least some of these mitochondria were capable of ATP production despite having been outside the cell and in the bloodstream, with aged mice averaging 364.4 fmoles of ATP production per sample (*n* = 3) and young mice averaging 18 fmoles per sample (*n* = 3; Figure 3h).

### 2.4. Result Summary

These results show several different applications of FAMS including mitophagy assays, EV detection, and the collection of cell-free mitochondria from blood plasma. See Table 1 for a brief overview of the goal, approach, and result for each FAMS application addressed above.

## 3. Discussion

Herein, we describe the parameters for the quantitative analysis of mitochondrial function by flow cytometry. First, we established a quantitative assay for the assessment of mitophagy, independent from chemical uncoupling leading to the dissipation of ΔΨ_M_. In this assay, we showed that mitochondria targeted for mitophagy can be evaluated based on the tandem expression of the molecular identifiers PINK1 and Parkin and further analyzed in downstream applications, such as mitochondrial DNA analysis. While the role of PINK1 and Parkin in mitophagy is now broadly accepted, there have been specific limitations to this body of work that have hindered our understanding of this innate process, namely, the reliance on high concentrations of the protonophores CCCP and FCCP, which dissipate ΔΨ_M_ through uncoupling of the electron transport chain. Importantly, these agents have also been shown to depolarize additional cellular compartments, including the lysosome, and lead to acidification of the cytosol, all of which impact mitochondrial function and the process of mitophagy [28]. Based on these limitations, physiologically relevant model systems and more precise tools have been called for in the study of mitochondrial function and, in particular, mitophagy [10]. In the assay we described for the quantitative assessment of mitophagy in individual mitochondria, we purposefully omitted the use of protonophores, which we have described previously [1], and instead opted for the use of a model in which mitophagy is naturally elevated: the PolG mutant mouse. Using this model, we were able to validate this assay, which is more broadly applicable across cell and tissue types, for the quantitative study of mitophagy. Utilizing this flow cytometric assay for the identification of mitochondria targeted for mitophagy will enable researchers to identify subtle inductions of this critical mitochondrial degradation pathway in response to treatment and disease states, as well as isolate and perform downstream assays on this organelle subpopulation.

Next, we described methodological details for the detection of mitochondria within secreted EVs. Macrophages are known for EV secretion in vitro and in vivo [29,30], and combining our FAMS strategy with the antibody-based detection of CD63+ permitted the reliable identification of dual-labeled events that were representative of mitochondria contained within EVs. Notably, many of these mitochondria maintained ΔΨ_M_. While it is still to be determined whether this phenomenon occurs in vivo, one potential function could be signaling or resource sharing by these mitochondria if they are internalized into other cells through the process of mitochondrial transfer [31,32]. Given that the rate of excretion and half-life of these mitochondria-containing EVs is currently unknown, it is also possible that the detected mitochondria that were not colocalized with CD63 may have resulted from the EVs degrading and releasing the mitochondria into the media. This theory is supported by the ΔΨ_M_, where mitochondria within vesicles have a high ΔΨ_M_ 96.3% (*n* = 3, SEM = 1.19) of the time compared to only 40.6% (*n* = 3, SEM = 14.9, *p* = 0.02) of mitochondria found outside of vesicles. Once the vesicles degrade and release the mitochondria into the media, their ΔΨ_M_ begins to drop. Ultimately, the excretion of these mitochondria does not appear to be random, especially given that ΔΨ_M_ is often maintained, which is indicative of active respiration.

Finally, we demonstrated the utility of FAMS to analyze and isolate mitochondria in circulation. Given that young individuals are known to have an increased amount of mtDNA carrying EVs in their plasma, which have a stronger effect on respiration rate compared to EVs from aged individuals [27], it is interesting that sorted mitochondria from the plasma of aged individuals showed higher ATP production than those from young individuals. Assuming mtDNA abundance correlated with intact organelle abundance in plasma, it is possible that the cell-free mitochondria of aged individuals produce more ATP in an attempt to compensate for their decreased abundance. Regardless of the observed age-associated differences, these findings confirm the presence of intact, respiratory-competent mitochondria in blood plasma. While the physiological purpose (if any) and origin of these cell-free mitochondria are unclear, their presence warrants further investigation into exactly what role they may be playing in the circulatory system. The potential for these organelles to act as signaling molecules or resource sharing between cells would be another addition to the increasing number of non-canonical mitochondrial functions currently being uncovered by new technologies such as FAMS.

In conjunction, these findings show that FAMS is a promising technique for studying non-canonical mitochondrial function. The implications of this technique on the field of mitochondrial research are clear, allowing researchers to detect and isolate specific mitochondrial subpopulations for study as opposed to taking aggregate readings from the heterogeneous mitochondrial population of a whole cell. With recent studies implicating mitochondria in phenomena such as stem cell differentiation [3,33,34], aging [7,35,36], and tumorigenesis [37,38,39], understanding the role of each mitochondrial population within the cell is paramount. While the data presented showcase the broad applicability of FAMS to study mitochondria and other organelles in great detail and non-standard contexts, the limitations of the technique should be noted. Firstly, cell lysis is required for single-organelle analysis, and this process removes mitochondria from their natural environment. Moving from the cytosol into a buffer and breaking away from the cytoskeleton could alter mitochondrial properties prior to analysis. Additionally, while not conducted in this manuscript, FAMS preparation for proteomics requires a large sample input. Typically, we sorted 2.2 × 10^7^ mitochondria for one proteomics sample prepared with a low-input protocol. The number of mitochondria required for this technique makes it unfeasible for many mitochondrial subpopulations that simply are not abundant enough to meet that threshold.

The data presented in this publication demonstrate the potential for FAMS to produce significant contributions to the study of mitochondria outside of their traditional role as energy producers within the cell, as well as the ability to analyze other organelles of a similar size with only minor adjustments. The ability to analyze individual, intact mitochondria on the basis of characteristics such as size, ΔΨ_M_, and protein expression allows for a quantitative measure of the mitochondrial heterogeneity that exists within a single cell type. Another advantage of FAMS is its versatility in terms of starting material. Previous work has been largely relegated to the study of mitochondria within cells, while only recently reports such as free-floating mitochondria in the blood have begun to explore the phenomenon of mitochondria being present outside the cell and what potential purpose they may serve [25,26]. FAMS is not only capable of detecting and analyzing such obscure mitochondrial populations but also sorting them in a manner that retains any respiratory capacity they may have, allowing for the downstream characterization of energy production in addition to other characteristics such as mtDNA copy number and protein content. Looking to the future of mitochondrial research, innovative techniques such as FAMS will be key to elucidating the diverse array of mitochondrial functions posed in recent years.

## 4. Materials and Methods

### 4.1. Animals and Cell Lines

All experiments described herein were reviewed and approved by the Institutional Animal Care and Use Committee of Northeastern University. For experiments involving blood collection, female C57BL/6 mice from Jackson Laboratories were used. Blood was collected from both young (13 weeks old) and aged (18 months old) mice. Approximately 1.25 mL of blood per mouse was collected via retro-orbital bleeding.

For the generation of fibroblast cell lines, WT female C57BL/6 and female *PolG* mutant mice that harbor a D257A mutation in the exonuclease domain of *PolG* were used. To generate the *PolG* ^D257A/D257A^ mouse line, heterozygous mutant male mice (*PolG* ^D257A/+^) obtained from the Jackson Laboratory were mated with WT C57BL/6 females to ensure a WT start point for the maternally inherited mitochondrial genome. *PolG*^D257A/+^ breeding pairs were then used to generate the homozygous knock-in mtDNA mutator mice (*PolG*^D257A/257A^). Fibroblast lines were then generated from the tail tips of both wild-type (C57BL/6) and *PolG* mutant mice by removing and skinning their tail tips before placing them in a gelatinized 10 cm plate containing 15 mL of culture media (10% FBS, High Glucose DMEM with sodium pyruvate, Pen/Strep, 2-ME). Tissue was then minced and allowed to expand for 5 days at 37 °C and 5% CO_2_. Cells were immortalized via transduction of an SV40 plasmid (Addgene 13970, Watertown, MA, USA) using the VSV-G vector (Addgene 14888, Watertown, MA, USA). Fibroblast cell lines were then maintained using DMEM (Fisher, Waltham, MA, USA) with 10% FBS and passaged multiple times to ensure they were stable prior to use in any experiments.

For experiments using macrophages, human THP-1 monocytes were obtained from the ATCC (Manassas, VA, USA). THP-1 monocytes were cultured in a 10 cm plate. To generate macrophages, phorbol 12-myristate-13-acetate was added to the culture at a concentration of 100 nM, and cells were allowed to differentiate into macrophages over the course of 48 h. This differentiation process also caused the cells to become adherent during this time. Both THP-1 macrophages and monocytes were maintained using RPMI 1640 (Fisher, Waltham, MA, USA) with 10% FBS.

### 4.2. Sample Generation

#### 4.2.1. From Blood (Cell-Free Mitochondria)

Blood collection via retro-orbital bleeding was performed into an Eppendorf tube containing 125 μL of buffered 3.2% sodium citrate in water, which assumes roughly a 1:9 ratio of buffer to blood for a final volume of approximately 1.25 mL. Blood samples were centrifuged at 200× *g*, 200× *g*, and 300× *g* consecutively for 10 min per spin, collecting the supernatant each time to isolate the blood plasma. Pre-warmed (37 °C) ACD-A buffer (Sigma-Aldrich, St. Louis, MO, USA) was added at a 1:6 buffer-to-plasma ratio. To this suspension, prostaglandin E1 (Fisher, Waltham, MA, USA) was added at a final concentration of 1 μM. Samples were then centrifuged at 1100× *g* for 10 min, and the resulting supernatant was collected, while the pellet was discarded. Samples were then centrifuged at 2500× *g* for 10 min to remove any remaining whole cells, and the supernatant was once again collected. Mitochondria were isolated from plasma by centrifugation at 16,000× *g* for 10 min at 4 °C. The supernatant was removed, and the samples were resuspended in 1 mL of PEB buffer (PBS containing 2 mM EDTA and 0.5% bovine serum albumin (BSA)) and split between four tubes (unstained, two single-color stains, and an all-stain tube). Designated samples were stained with MitoTracker™ Green FM (MTG; Life Technologies, Carlsbad, CA, USA) at 100 nM and JC-1 (Fisher, Waltham, MA, USA) at 1 μM and incubated for 15 min at room temperature. Following incubation, samples were centrifuged at 12,000× *g* for 5 min at 4 °C. The supernatant was discarded, and the samples were washed with 250 μL of the PEB buffer and centrifuged again at 12,000× *g* for 5 min at 4 °C. The supernatant was discarded, and the samples were resuspended in 300 μL of a sheath buffer (blood-bank saline) and strained through a 70 μm filter (Fisher) into polystyrene FACS tubes. The samples were then run on a BD FACSAriaIII (BD Biosciences, Franklin Lakes, NJ, USA). All samples analyzed were included in the results presented in this manuscript.

#### 4.2.2. From Plated Cells

Plated immortalized mouse fibroblast cells were grown in 10 cm plates to near confluency (approximately 6 × 10^6^ cells). The cells were then collected and divided evenly between the sample tubes. Designated samples were stained with MTG at 100 nM, and JC-1 at 1 μM was incubated for 15 min at 37 °C. The samples were then centrifuged at 500× *g* for 5 min, and the supernatant was discarded. The samples were resuspended in an RSB-Hypo lysis buffer (10 mM NaCl, 1.5 mM MgCl2, 10 mM Tris-HCl (pH 7.6), and 1X Halt Protease Inhibitor (Fisher, Waltham, MA, USA) in water) and incubated at 4 °C for 10 min to allow for swelling and initiation of cell lysis. After cell swelling, the samples were dounce homogenized until >90% cell lysis was confirmed via a 1 μL droplet taken and observed with a microscope (typically ~35 strokes). Samples were then centrifuged at 12,000× *g* for 5 min. The supernatant was discarded, and the samples were resuspended in 1 mL of a blocking buffer (2% BSA in PBS) for 20 min at room temperature. The samples were then centrifuged at 12,000× *g* for 5 min, followed by resuspension in the appropriate antibody solution (Parkin (Abcam, ab77924, Cambridge, UK) and/or PINK1 (Abcam, ab2370, Cambridge, UK 7) and directly conjugated with APC-Cy7 (Abcam, ab102859, Cambridge, UK) or APC (Abcam, ab201807, Cambridge, UK), respectively, while the remaining samples were resuspended in a blocking buffer. All samples were then incubated for 20 min at room temperature. Samples were then centrifuged at 12,000× *g* for 5 min at 4 °C and washed with a PEB buffer 2x and then resuspended in 1 mL of sheath fluid and strained through a 70 μm filter into a polystyrene FACS tube. The samples were then run on a BD FACSAriaIII (BD Biosciences, Franklin Lakes, NJ, USA).

#### 4.2.3. From Cell Culture Media

THP-1 monocytes in 10 mL of RPMI 1640 (Fisher, Waltham, MA, USA ) + 10% FBS in a 10 cm plate were incubated at 37 °C and 5% CO_2_ for 24 h prior to media collection. Spent media were collected and divided between sample tubes. The samples were centrifuged at 500× *g* for 5 min to pellet out any whole cells. The supernatant was collected, and the designated samples were stained with 100 nM MitoTraker Green and 1 μM JC-1, followed by a 15 min incubation at 37 °C. Following staining, the samples were centrifuged at 12,000× *g* for 5 min at 4°C. The supernatant was discarded, and the samples were resuspended in 1 mL of blocking buffer for 20 min at room temperature. Following blocking, the samples were centrifuged at 12,000× *g* for 5 min, and the samples were resuspended in antibody solution (APC-conjugated anti-CD63; Abcam, ab8219, Cambridge, UK) or in a blocking buffer for 20 min at room temperature. The samples were then centrifuged at 12,000× *g* for 5 min at 4 °C and washed with 1 mL PEB buffer. The samples were centrifuged again and resuspended in 1 mL of sheath fluid before being strained through a 70 μm filter. The samples were then run on a BD FACSAriaIII (BD Biosciences, Franklin Lakes, NJ, USA).

### 4.3. Flow Cytometric Analysis and Sorting

**Figure 4:**
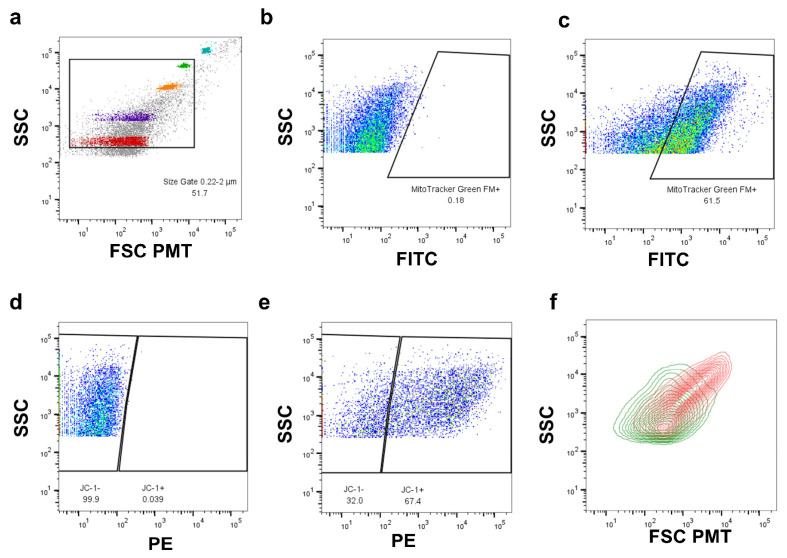
Demonstrative example of FAMS gating tree showing specific mitochondrial subpopulations. (**a**) Using a FSC-PMT diode detector and voltages optimized for sub-cellular sorting, an initial size gate was drawn from 0.22 μm to 2.0 μm to isolate events within the size range of mitochondria from the ungated sample. This size range was established in accordance with studies on mitochondrial size using electron microscopy [1]. To determine this size range on the flow cytometer, size-calibrated beads (0.22 μm [red], 0.45 μm [purple], 1 μm [orange], 2 μm [green], and 4 μm [blue]) were recorded immediately prior to the first sample [gray] and were used to generate the initial size gate. (**b**) An unstained sample was used as a negative control to gate for MitoTracker Green FM (MTG) in the FITC channel, which was used to label mitochondria within the sample. (**c**) MTG-positive events are shown, comprising 61.5 percent of events that initially fell within the size gate. Values similar to this are to be expected, as prior centrifugation steps during sample preparation enrich for mitochondria. MTG allowed for the identification of mitochondria within the sample and the exclusion of any debris that happened to fall within the mitochondrial size gate. (**d**) A sample stained with MTG but not JC-1 was used to establish JC-1 gating. JC-1 shifts from the FITC channel to the PE channel upon aggregate formation, which occurs within mitochondria with a high ΔΨ_M_ but not those with a low ΔΨ_M_. Events that do not fall within the size gate or are not MTG-positive were excluded from JC-1 consideration. (**e**) Determination of mitochondrial membrane potential using JC-1 staining. In this instance, 67.4 percent of mitochondria in this sample were shown to have a high ΔΨ_M_. (**f**) Size overlay of high (red) and low (green) ΔΨ_M_ mitochondria. This overlay shows an example comparison of two mitochondrial subpopulations, in this case showing that high-membrane-potential mitochondria tend to be larger, while low-membrane-potential mitochondria tend to be lower in size.

Machine startup and laser calibration using fluorescent rainbow beads (556291, Fisher, Waltham, MA, USA) were run. The sub-micron mitochondrial size range was established using fluorescent size beads (0.22 and 0.45 μm; NC0483352, Fisher, Waltham, MA, USA) to distinguish beads from noise using the FSC channel with a photomultiplier tube to boost signal definition in the sub-micron size range. Non-fluorescent size beads (1, 2, and 4 μm; F13838 LifeTech, Carlsbad, CA, USA) were used to establish the upper range in mitochondrial size (Figure 4a). Mitochondria were identified and gated based on size and FITC fluorescent MitoTracker Green labeling compared to unstained controls (Figure 4b,c). Additional fluorescence gates were then subsequently applied for any further markers used, such as JC-1, that show ΔΨ_M_ (Figure 4d,e). Next, mitochondria with different characteristics or protein expression, as determined by these analyses, were defined into gated subpopulations and compared to one another; in this example, the sizes of high and low ΔΨ_M_ mitochondria were compared (Figure 4f). For all analyses, a minimum of 3 × 10^4^ events were recorded per sample. In cases where fluorophores may have some spill over into other active channels, such as APC and APC-Cy7 for the plated cells analysis, compensation was run to ensure a clean signal in both channels.

Analysis was conducted using a special-order research product, BD FACS Aria III, equipped with a photomultiplier tube detector for the forward light scatter (FSC) of a 488 nm laser, allowing for an increased dynamic range of small particle detection over standard photodiode detectors. In order to detect events in the size range of mitochondrial (~0.2–2 μm), the threshold for detection was set to side light scatter (SSC) 200 [1]. Data were acquired using BD FACSDiva software (version 8.0.2) and then further analyzed using FlowJo (v10.10.0) and Microsoft Excel (v16.61.1). To sort mitochondria, instrument calibration was performed using BD FACSDiva “Accudrop” and “test sort” procedures. “Accudrop” was performed using accudrop beads (BD) for all sorts. For single mitochondria sorts into 96-well plates, “test sort” was performed with the “single-cell” purity mask selected to ensure sample purity. For sorting larger quantities of mitochondria, “test sort” was performed into a 2.0 mL Eppendorf tube with the “4-way purity” mask selected.

### 4.4. Downstream Applications

#### 4.4.1. Single-Molecule PCR (smPCR)

To each well of a 96-well plate, 1 µL of a mitochondrial lysis buffer (10 mM EDTA, 0.5% SDS, and 0.1 mg/mL proteinase K in water) was added. After the sort calibration had taken place, the plate was loaded into the flow cytometer. Single mitochondria were sorted into each well of the 96-well plate using the “single cell” purity mask, as described above. Once the sorting was complete, all wells were immediately overlayed with mineral oil (Sigma-Aldrich, St. Louis, MO, USA ) to prevent evaporation and were incubated at 37 °C for 1 h to allow for lysis of the mitochondria. The plate was then transferred to a −80 °C freezer until preparation for a single-molecule polymerase chain reaction (smPCR). To perform smPCR, lysed samples were each diluted across 32 wells of a 96-well plate in order to greatly decrease the possibility of multiple mtDNA copies ending up in the same well, in addition to diluting the mitochondrial lysis buffer to the point that it will not negatively impact DNA replication. This dilution was performed with the PCR replication mixture of Ex Taq DNA Polymerase, Hot Start Version (Takara, San Jose, CA, USA) with an LA PCR buffer (Takara, San Jose, CA, USA) to generate 331 base pair amplicons. For primer information, please see Macdonald et al. [1]. Following gel electrophoresis, the mtDNA copy number was determined by manually counting the number of bands present in each 32-well sample, as the smPCR method has been optimized in such a way that each band represents one mtDNA copy. The results were analyzed using GraphPad Prism (v10.4.1).

#### 4.4.2. ATP Assay

Using “4-way” purity as described above, 1 × 10^6^ mitochondria were sorted into a 2 mL Eppendorf tube. Sorted mitochondria were centrifuged at 12,000× *g* and resuspended in a mitochondrial respiration buffer (225 mM D-mannitol, 75 mM sucrose, 10 mM KCl, 10 mM Tris-HCl, and 5 mM KH2PO4, pH 7.2 in water). ATP standards and luciferase reagents were prepared from a standard ATP bioluminescence assay kit (Sigma-Aldrich, St. Louis, MO, USA). Luminescence was analyzed immediately following the addition of the luciferase reagent to the assay (emission ~560 nm). In this experiment, a Biotek Synergy H1 plate reader was used. ATP standards were used to generate a standard curve, which, by comparison, allows for the determination of ATP production by the samples.

## Figures and Tables

**Table 1:** This table summarizes the results section.

Experimental Aim	Experimental Approach	Result
Development of a biological assay for the quantitative assessment of mitophagy in individual mitochondria without the use of membrane uncouplers.	Pol G mutant fibroblast mitochondria were assessed for ΔΨ_M_, PINK1, and Parkin expression using FAMS. Individual mitochondria were sorted and assessed for mtDNA copy number.	PINK1 and Parkin expression was detected on a per organelle basis without the use of membrane uncouplers and were shown to colocalize at different rates based on mitochondrial subpopulation.
Assessment of spent cell culture media to determine whether detected cell-free mitochondria were free-floating or contained within extracellular vesicles.	Following a 24 h incubation of THP-1 macrophages, spent media were collected, ensured to be free of cells, and assessed for the presence of free mitochondria and extracellular vesicles using FAMS.	Both free-floating and vesicle-enclosed mitochondria were detected. The detection of vesicle-enclosed mitochondria raises the question of whether these organelles are being intentionally excreted by the macrophages in vitro.
Test for the presence of intact, cell-free mitochondria in mouse blood plasma.	Plasma was collected and tested for the presence of respiratory-competent mitochondria using FAMS. Sorted mitochondria were pooled and tested for the ability to produce ATP.	Cell-free, respiratory-competent mitochondria were detected in mouse plasma using FAMS. Their ability to respire was confirmed by ATP assay.

## Data Availability

The data presented in this study are included in the article, and further inquiries can be directed to the corresponding author.

## Abbreviations

The following abbreviations are used in this manuscript:

FAMS	Fluorescence-activated mitochondria sorting
ΔΨ_M_	Mitochondrial membrane potential
mtDNA	Mitochondrial DNA
ROS	Reactive oxygen species
EV	Extracellular vesicle
PolG	Polymerase gamma
MTG	MitoTracker Green FM
PINK1	Phosphatase and tensin homolog (PTEN)-induced putative kinase 1
smPCR	Single-molecule PCR
SEM	Standard error of the mean
